# Characteristics and outcomes of cancer patients in European ICUs

**DOI:** 10.1186/cc7713

**Published:** 2009-02-06

**Authors:** Fabio Silvio Taccone, Antonio A Artigas, Charles L Sprung, Rui Moreno, Yasser Sakr, Jean-Louis Vincent

**Affiliations:** 1 Department of Intensive Care, Erasme Hospital, Université libre de Bruxelles, Route de Lennik 808, 1070-Brussels, Belgium; 2 Critical Care Center, Sabadell Hospital, CIBER Enfermedades Respiratorias, Autonomous University of Barcelona, Parc Tauli, 08208 Sabadell, Spain; 3 Department of Anesthesiology and Critical Care Medicine, Hadassah Hebrew University Medical Center, P.O.B. 12000, 91120 Jerusalem, Israel; 4 Department for Intensive Care, Hospital de St. Antonio dos Capuchos, Centro Hospitalar de Lisboa Central E.P.E., Alameda de Santo António dos Capuchos, 1169-050 Lisboa, Portugal; 5 Department of Anesthesiology and Intensive Care, Friedrich-Schiller-University, Erlanger Allee 101, Jena 07743, Germany

## Abstract

**Introduction:**

Increasing numbers of cancer patients are being admitted to the intensive care unit (ICU), either for cancer-related complications or treatment-associated side effects, yet there are relatively few data concerning the epidemiology and prognosis of cancer patients admitted to general ICUs. The aim of this study was to assess the characteristics of critically ill cancer patients, and to evaluate their prognosis.

**Methods:**

This was a substudy of the Sepsis Occurrence in Acutely Ill Patients (SOAP) study, a cohort, multicentre, observational study that included data from all adult patients admitted to one of 198 participating ICUs from 24 European countries during the study period. Patients were followed up until death, hospital discharge or for 60 days.

**Results:**

Of the 3147 patients enrolled in the SOAP study, 473 (15%) had a malignancy, 404 (85%) had solid tumours and 69 (15%) had haematological cancer. Patients with solid cancers had the same severity of illness as the non-cancer population, but were older, more likely to be a surgical admission and had a higher frequency of sepsis. Patients with haematological cancer were more severely ill and more commonly had sepsis, acute lung injury/acute respiratory distress syndrome, and renal failure than patients with other malignancies; these patients also had the highest hospital mortality rate (58%). The outcome of all cancer patients was comparable with that in the non-cancer population, with a 27% hospital mortality rate. However, in the subset of patients with more than three failing organs, more than 75% of patients with cancer died compared with about 50% of patients without cancer (p = 0.01).

**Conclusions:**

In this large European study, patients with cancer were more often admitted to the ICU for sepsis and respiratory complications than other ICU patients. Overall, the outcome of patients with solid cancer was similar to that of ICU patients without cancer, whereas patients with haematological cancer had a worse outcome.

## Introduction

Remarkable advances have been made in the early diagnosis and aggressive management of patients with malignancies, resulting in dramatic improvements in overall survival rates [1,2]. As a result, increasing numbers of patients are admitted to the intensive care unit (ICU), either for cancer-related complications or for treatment-associated side effects [3]. Several studies have reported very high mortality rates for cancer patients after a long ICU stay, especially when they had leucopenia [4] or required mechanical ventilation [5], and aggressive management of life-threatening complications in these patients has been questioned [6]. In a prospective, longitudinal study performed in 26 ICUs, Azoulay and colleagues found that cancer patients were at a high risk of being denied ICU admission [7], in accordance with articles discouraging ICU admission or prolonged intensive care for cancer patients [6,8]. However, other studies have highlighted reduced mortality rates in critically ill patients with cancer [9,10], and the development of new procedures, such as non-invasive mechanical ventilation, may enable recommendations for ICU admission and appropriate utilisation of ICU resources for cancer patients to be altered [11].

Several large epidemiological studies have provided findings on prognostic factors for cancer patients admitted to the ICU [1,12,13], but these studies essentially concerned specialised oncological ICUs, so extrapolation to general ICUs and hospitals can be difficult. There are several issues of particular interest. First, it is important to determine if mortality rates are different for patients with and without cancer in a general ICU. In particular, because sequential assessment of organ failure is fundamental to predict outcome in the general ICU population [14], it would be interesting to know whether the relationship between the number of acute organ failures and mortality is different in patients with and without malignancy. Second, sepsis remains one of the major causes of admission for cancer patients to the ICU and is an important cause of hospital mortality and morbidity [15]. Moreover, treatment of cancer has contributed to a growing number of immunocompromised patients with an increased incidence of nosocomial infections [16]; immunosuppression can result in a greater use of antibiotics and more infections associated with multiresistant micro-organisms [17]. It is, therefore, also important to define whether cancer patients have more sepsis episodes and sepsis-related organ dysfunctions than non-cancer patients.

The Sepsis Occurrence in Acutely Ill Patients (SOAP) study [15] collected a large amount of data on all patients admitted to general (non-specialised) ICUs during a two-week period. As there are relatively few data concerning the epidemiology and prognosis of cancer patients admitted to general ICUs or the epidemiology and patterns of sepsis syndromes in these patients [17,18], the aim of this study was to assess the characteristics of critically ill cancer patients, and to evaluate their prognosis.

## Materials and methods

### Study design

This study was a substudy of the prospective, multi-centre, observational SOAP study. The SOAP study [15] was designed to evaluate the epidemiology of sepsis and to identify various aetiological, diagnostic, therapeutic and prognostic factors of ICU patients in European countries, and was endorsed by the European Society of Intensive Care Medicine. Although this observational study did not require any deviation from routine medical practice, institutional review board approval was either waived or expedited in participating institutions and informed consent was not required. As such, no supplementary review board documents were needed for the current sub-study.

All patients older than 15 years that were newly admitted to the ICU of a participating centre (see the list of participating countries and centres in Additional File 1) between 1 and 15 May, 2002, were included in the study. Patients were followed up until death, hospital discharge or for 60 days, whichever came first. Those who stayed in the ICU for longer than 24 hours for routine postoperative observation were excluded. Patients who were readmitted and had been included on their first admission were not included for a second time.

### Definitions

Details of all the definitions used in the SOAP study have been published previously [15]. Infection was defined as the presence of a pathogenic micro-organism in a sterile site (such as blood, abscess fluid, cerebrospinal fluid or ascites) and/or clinically suspected infection, plus the administration of antibiotics. Sepsis was defined according to standard criteria [19]. ICU-acquired sepsis was defined as sepsis occurring more than 48 hours after admission to the ICU. Patients were defined as having acute lung injury (ALI) or acute respiratory distress syndrome (ARDS) if the arterial oxygen pressure to inspiratory oxygen fraction ratio (PaO_2_/FiO_2_) was less than 300 for ALI and less than 200 for ARDS and all of the following were present: bilateral infiltrates on the chest radiograph; no clinical evidence of heart failure; no chronic pulmonary disorders; and mechanical ventilation. Organ failure was defined as a Sequential Organ Failure Assessment (SOFA) score more than 2 for the organ in question [20]. Patients were classified as surgical admissions if they had undergone surgery within two weeks preceding admission.

Cancer was identified as solid or haematological malignancy diagnosed before admission to the ICU. For solid tumours, the presence of metastases was also recorded. Patients with a prior history of cancer and with complete remission for over five years were not considered in the cancer group. Leucopenia was defined as a white blood cell count less than 1000 cells/mm^3^, and severe thrombocytopenia by a platelet count less than 50,000 cells/mm^3 ^[19].

### Data management

Data were collected prospectively using pre-printed case report forms filled in following instructions available on a dedicated website. The steering committee was easily accessible to the investigators and processed all queries during data collection. Data collection on admission included demographic data and comorbid diseases. Clinical and laboratory data for the Simplified Acute Physiology Score (SAPS) II [21] were reported as the worst value within 24 hours after admission. Microbiological and clinical infectious data were reported daily as well as the antibiotics administered. A daily evaluation of organ function based on the SOFA score [14] was performed, with the most abnormal value for each of six organ systems (respiratory, renal, cardiovascular, hepatic, coagulation and neurological) being collected on admission and every 24 hours thereafter. Data collection and quality control are described elsewhere [15].

### Statistical analysis

Data were analysed using SPSS 13.0 for Windows (SPSS Inc., Chicago, IL, USA). Descriptive statistics were computed for all study variables. A Kolmogorov-Smirnov test was used, and histograms and normal-quantile plots were examined to verify the normality of distribution of continuous variables. Discrete variables are expressed as counts (percentage) and continuous variables as means ± standard deviation (SD) or median (25th to 75th percentiles). For demographics and clinical characteristics of the study groups, differences between groups were assessed using a chi-square, Fisher's exact test, Student's t-test or Mann-Whitney U test, as appropriate.

Multivariate logistic regression analysis with hospital mortality as the dependent variable was conducted in patients with solid and haematological cancer. Only variables associated with a higher risk of hospital mortality (p < 0.25) on a univariate basis were introduced in the multivariate model. Colinearity between variables was excluded prior to modelling. A Hosmer-Lemeshow goodness-of-fit test was performed and Nagelkerke pseudo r^2^, classification tables, and odds ratios (OR) with 95% confidence interval (CI) were computed. Variables considered in the analysis were, therefore, demographic variables, co-morbidities, SAPS II score on admission, organ failure as assessed by the SOFA score on admission, presence of metastases, type of admission (medical or surgical), reason for admission, sepsis, source of infection, type of micro-organism (*Pseudomonas aeruginosa*, methicillin-resistant *Staphylococcus aureus*, *Escherichia coli*, *Candida *species) following results of descriptive data on infection incidence, mechanical ventilation, renal replacement therapy (haemofiltration or haemodialysis), administration of inotropes and/or vasopressor agents, presence of leucopenia, thrombocytopenia, ALI or ARDS. Kaplan-Meier survival curves were plotted and compared using a signed log-rank test. All statistics were two-tailed and a p < 0.05 was considered significant.

## Results

### Demography

From 3,147 patients enrolled during the study period, 473 (15%) had a malignancy. Of these, 69 (15%) had haematological cancer and 404 (85%) had solid tumours, of whom 100 had evidence of metastases. The patients with solid tumours were older than the patients without cancer and were more commonly male (Table 1). Surgical admissions accounted for almost 70% of the patients with solid cancer compared with 41% of those without cancer, and 20% of those with haematological cancer (Table 1). Gastrointestinal, thoracic, and renal/urological surgery were more common, and cardiovascular and neurosurgery less common, in patients with solid tumours than in those without cancer. Cancer patients were more commonly admitted for respiratory reasons, but less commonly for acute neurological diseases and trauma. SAPS II and SOFA scores were comparable in patients with solid tumours and those without cancer, but both scores were significantly higher in patients with haematological cancer than in those without cancer. The median lengths of stay in the ICU were quite similar in the three groups, but cancer patients had longer hospital stays than those without cancer. Co-morbidities were different among the groups, with a lower prevalence of cardiac insufficiency in patients with solid tumours, and more patients with AIDS in patients with haematological cancer. Corticosteroids and chemotherapy were more commonly used in patients with cancer than in those without.

**Table 1:** Demographic characteristics of patients

	**No cancer** **(n = 2674)**	**Solid tumours** **(n = 404)**	**Haematological cancers** **(n = 69)**
**Age, years**	59.6 ± 17.9	66.4 ± 12.1^$^	62.1 ± 15.9
**Male^a^**	1619 (61.2%)	265 (66.6%)*	36 (52.9%)
**Type of admission**			
**Medical**	1581 (59.1%)	123 (30.4%)^$^	55 (79.7%)*
**Surgical**	1093 (40.9%)	281 (69.6%)^$^	14 (20.3%)*
Neurosurgery	131 (11.9%)	20 (7.1%)*	1 (7.1%)
Digestive surgery	284 (26.0%)	174 (61.9%)^$^	7 (50%)
Thoracic surgery	28 (2.5%)	24 (8.5%)^$^	2 (14.2%)
Cardiovascular surgery	453 (41.4%)	13 (4.6%)^$^	3 (21.4%)
Renal/urological surgery	25 (2.3%)	22 (7.8%)^$^	0
Other surgery	136 (12.4%)	23 (8.2%)*	1 (7.1%)
**Admission source**			
Hospital floor	639 (26.4%)	118 (33.1%)*	36 (61.0%)^$^
ER/ambulance	849 (35.1%)	56 (15.7%)^$^	8 (13.6%)^$^
Recovery room	623 (25.7%)	152 (42.7%)^$^	9 (15.3%)
Other hospital	309 (12.8%)	30 (8.4%)*	6 (10.2%)
**Reason for admission**			
Surveillance	192 (7.6%)	54 (14.8%)^$^	1 (1.4%)
Digestive/liver	236 (9.3%)	88 (24.1%)^$^	9 (13.0%)
Respiratory	432 (17.0%)	96 (26.3%)^$^	32 (46.4%)^$^
Cardiovascular	874 (34.5%)	56 (15.3%)^$^	19 (27.5%)
Haematological	24 (0.9%)	3 (0.8%)	0
Neurological	446 (17.6%)	36 (9.9%)^$^	3 (4.3%)*
Renal	81 (3.2%)	19 (5.2%)	4 (5.8%)
Metabolic	60 (2.4%)	10 (2.7%)	1 (1.4%)
Trauma	179 (7.1%)	2 (0.5%)^$^	0*
**Comorbidities and therapies on admission**			
COPD	292 (10.9%)	42 (10.4%)	6 (8.7%)
Diabetes	200 (7.5%)	24 (5.9%)	2 (2.9%)
Liver cirrhosis	103 (3.9%)	18 (4.5%)	0
AIDS	12 (0.4%)	3 (0.7%)	3 (4.3%)*
Heart failure	276 (10.3%)	22 (5.4%)*	9 (13%)
Corticosteroids	123 (4.6%)	28 (6.9%)*	14 (20.3%)^$^
Chemotherapy	8 (0.3%)	10 (2.5%)^$^	7 (10.1%)^$^
**SAPS II**	36.0 ± 16.8	36.8 ± 17.6	53.5 ± 18.5^$^
**Incidence of sepsis**			
Sepsis	960 (35.9%)	168 (41.5%)^$^	49 (71%)^$^
Severe sepsis	780 (29.1%)	110 (27.2%)	40 (57.9%)^$^
Septic shock	380 (14.3%)	57 (14.1%)	23 (33.3%)^$^
Sepsis on admission	634 (23.7%)	107 (26.5%)	36 (52.2%)^$^
ICU-acquired sepsis	228 (8.5%)	43 (10.6%)	8 (11.6%)
Severe sepsis on admission	462 (17.3%)	64 (15.8%)	26 (37.7%)^$^
Septic shock on admission	197 (7.4%)	31 (7.7%)	15 (21.7%)^$^
**Admission SOFA**	5.2 ± 3.8	4.6 ± 3.6	7.0 ± 4.6*
**ICU stay, days**	3.0 (1.7 to 7.0)	3.0 (1.8 to 6.4)	3.8 (1.7 to 8.6)
**Hospital stay, days**	14.0 (7.0 to 31.0)	20.0 (12.0 to 33.0)*	22.5 (10.0 to 38.0)*

COPD = chronic obstructive pulmonary disease; ER = emergency room; ICU = intensive care unit; SAPS = simplified acute physiology score; SOFA = sequential organ failure assessment. Data are presented as mean ± standard deviation, number (percentage), or median (interquartile range).

* p < 0.05 versus no-cancer group; $ p < 0.001 versus no-cancer group; ^a ^35 missing values

### Frequency, distribution and patterns of sepsis

Of 1,177 (37% of the total population) patients with identified infection, 217 (18%) had cancer (Table 1). More patients with haematological cancer had severe sepsis and septic shock than patients without cancer, already on admission. There was no difference in the rate of ICU-acquired infections among the three groups. The most common site of infection in all three groups, both at admission and during the ICU stay, was the lung (Table 2). Abdominal infections occurred more frequently in patients with solid cancer compared with patients without cancer. Patients with haematological cancer had more episodes of bacteraemia than patients without cancer. The most common micro-organisms are presented in Table 2. *E. coli *was more frequently isolated in cancer patients than in patients without cancer. There was no significant difference in the micro-organisms recovered from blood cultures (data not shown).

**Table 2:** Characteristics of infected patients according to the type of malignancy

	**No cancer** **(n = 960)**	**Solid tumours** **(n = 168)**	**Haematological cancer** **(n = 49)**
**Criteria for infection**			
Clinically suspected	750 (78.1%)	136 (81%)	38 (77.6%)
Microbiologically confirmed	666 (69.4%)	114 (67.9%)	34 (69.5%)
Clinical signs and micro-organism	383 (39.9%)	71 (42.5%)	14 (28.6%)
**Source of infection**			
Respiratory	648 (67.5%)	108 (64.3%)	38 (77.6%)
Abdominal	200 (20.8%)	56 (33.3%)^$^	7 (14.3%)
Blood stream	196 (20.4%)	26 (15.5%)	16 (32.7)*
Skin	132 (13.8%)	23 (13.7%)	3 (6.1%)
Urinary	133 (13.9%)	22 (13.1%)	4 (8.2%)
Catheter	87 (9.1%)	18 (10.7%)	6 (12.2%)
Cerebrospinal fluid	15 (1.6%)	0 (0.0%)	0 (0.0%)
Unknown	53 (5.5%)	7 (4.1%)	3 (6.1%)
**Gram-positive bacteria**			
*Streptococcus group D*	97 (10.1%)	21 (12.5%)	5 (10.2%)
*Streptococcus pneumoniae*	42 (4.3%)	3 (1.7%)	1 (2.0%)
*MRSA*	131 (13.6%)	28 (16.6%)	5 (10.2%)
Other cocci	20 (2.1%)	3 (1.8%)	0
**Gram-negative bacteria**			
*Pseudomonas*	132 (13.7%)	21 (12.5%)	10 (20.4%)
*Escherichia coli*	114 (11.8%)	34 (20.2%)^$^	10 (20.4%)*
*Enterobacter*	53 (5.5%)	13 (7.7%)	1 (2.0%)
*Klebsiella*	60 (6.2%)	11 (6.5%)	0
*Proteus*	39 (4.0%)	9 (5.3%)	1 (2.0%)
*Acinetobacter*	37 (3.8%)	3 (1.7%)	2 (4.0%)
*Haemophilus*	33 (2.4%)	3 (1.8%)	1 (2.0%)
**Fungi**			
*Candida albicans*	125 (13%)	28 (16.7%)	3 (6.1%)
*Candida *non*-albicans*	37 (3.9%)	9 (5.4%)	3 (6.1%)
Other fungi	13 (1.3%)	3 (1.7%)	1 (2.0%)

MRSA: methicillin-resistant *Staphylococcus aureus*. *p < 0.05 versus no-cancer group; $ p < 0.001 versus no-cancer group. Data are presented as number (percentage).

### Organ dysfunction

**Figure 1:**
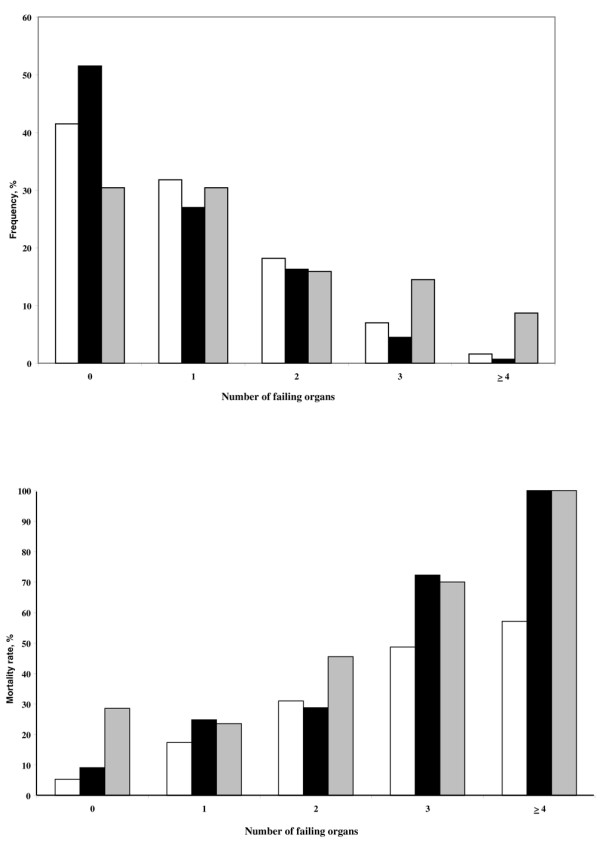
Organ dysfunction. Maximum number of organ dysfunctions during the intensive care unit (ICU) stay (upper panel) and hospital mortality according to the number of organ dysfunctions (lower panel) in the three groups of patients. White bars = no cancer; gray bars = haematological cancer; black bars = solid tumours.

Renal (29% versus 37%, p = 0.01) and neurological (20% versus 26%, p = 0.02) dysfunction were less common in patients with solid tumours than in those without cancer, and these differences were already present at admission. Patients with haematological cancer more commonly had respiratory (55% versus 40%, p = 0.01), circulatory (50% versus 32%, p = 0.001), and especially coagulation (45% versus 8%, p < 0.001) dysfunction during the ICU stay than patients without cancer. As expected, leucopenia was more common in patients with solid tumours and in patients with haematological cancer (Table 3). Patients with haematological cancer had lower PaO_2_/FiO_2 _ratios and a higher incidence of ALI/ARDS than patients without cancer. There were no differences in the number of failing organs per day (median 2.0 (interquartile range 1.0 to 3.0)) for the three groups; however, the mean number of organ failures was higher in patients with haematological cancer than in patients without cancer (p = 0.02). Figure 1 shows the number of organs failing and the corresponding mortality. Hospital mortality increased with the number of organs failing, especially in cancer patients when more than three organs failed (121 of 241 (50%) non-cancer patients versus 29 of 37 (78%) patients with cancer; p = 0.01).

**Table 3:** Respiratory and haematological dysfunction, ICU monitoring and treatment

	**No cancer** **(n = 2674)**	**Solid tumours** **(n = 404)**	**Haematological cancer** **(n = 69)**
**Mechanical ventilation**	1724 (64.5%)	253 (62.6%)	48 (69.6%)
**ALI/ARDS**	325 (12.2%)	47 (11.6%)	21 (30.4%)^$^
**PaO_2_/FiO_2_**	202.8 (133.4 to 295.0)	224.0 (144.0 to 324.3)	140.0 (94.0 to 206.2)^$^
**MV, days/patient**	3.0 (1.0 to 7.0)	2.0 (1.0 to 6.0)	4.0 (2.0 to 6.0)
			
**Leucopenia**	43 (1.6%)	14 (3.5%)*	17 (24.6%)^$^
**Thrombocytopenia**	373 (13.9%)	52 (12.9%)	35 (50.7%)^$^
			
**Pulmonary artery catheter**	430 (16.1%)	37 (9.2%)^$^	14 (20.3%)
**Central venous catheter**	1896 (70.9%)	317 (78.5%)	59 (85.5%)
**Arterial catheter**	1882 (70.4%)	304 (75.2%)	54 (78.3%)^$^
**Vasopressors**	1089 (40.7%)	163 (40.3%)	41 (59.4%)*
**Inotropes**	505 (18.9%)	61 (15.1%)	20 (29.0%)*
**Haemofiltration**	184 (6.9%)	16 (4.0%)	11 (15.9%)*
**Haemodialysis**	121 (4.5%)	16 (4.0%)	4 (5.8%)

ALI: acute lung injury; ARDS: acute respiratory distress syndrome; FiO_2_: inspired fraction of oxygen; ICU = intensive care unit; MV: mechanical ventilation; PaO_2_: arterial partial pressure of oxygen.

* p < 0.05 versus no-cancer group; $ p < 0.001 versus no-cancer group.

Data are presented as number (percentage) or median (interquartile range)

### Monitoring and therapy

Arterial catheters were more commonly used in patients with haematological cancer, but pulmonary artery catheters were less commonly used in patients with solid tumours (Table 3), and this difference was not explained by the type of surgery (cardiac surgery in particular) or the frequency of heart failure in a multivariable analysis (data not shown).

Mechanical ventilation was used in more than 60% of patients with similar median duration. Patients with haematological cancer were more often treated with haemofiltration, vasopressors and inotropes.

### Outcome

**Figure 2:**
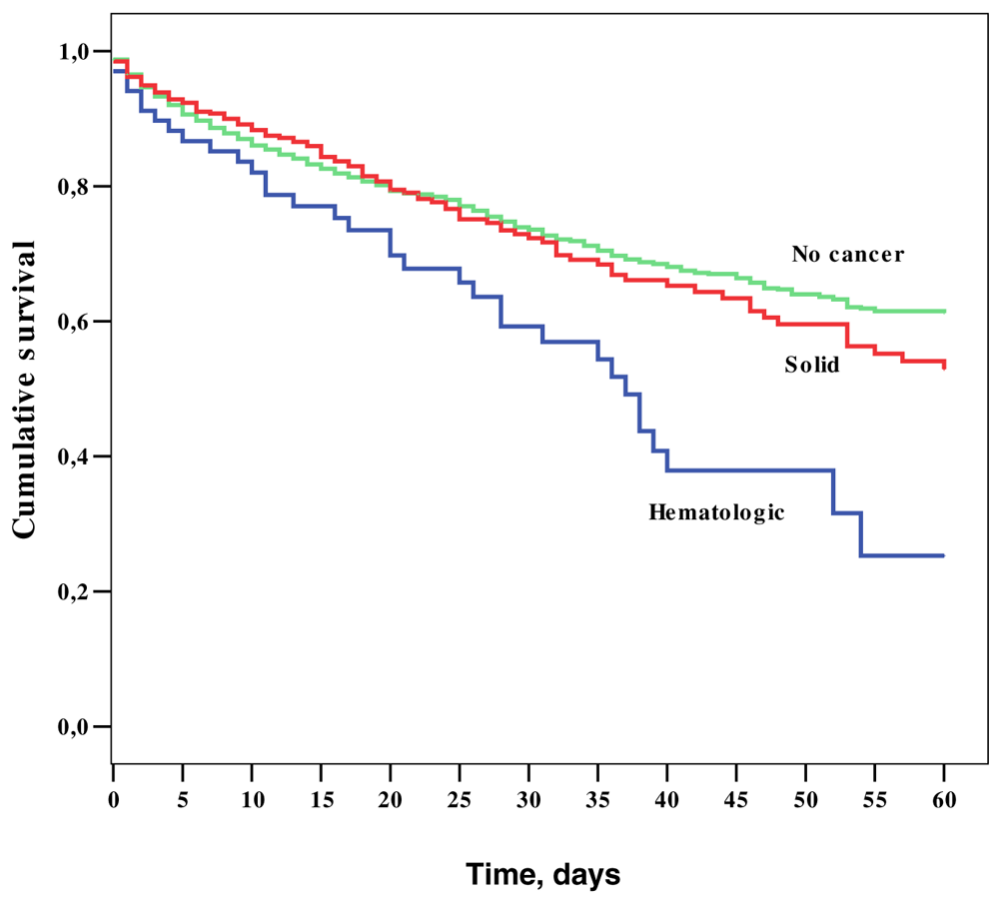
Kaplan Meier 60-day survival curves of the three groups of patients. Log Rank score = 20.78; p < 0.01.

**Figure 3:**
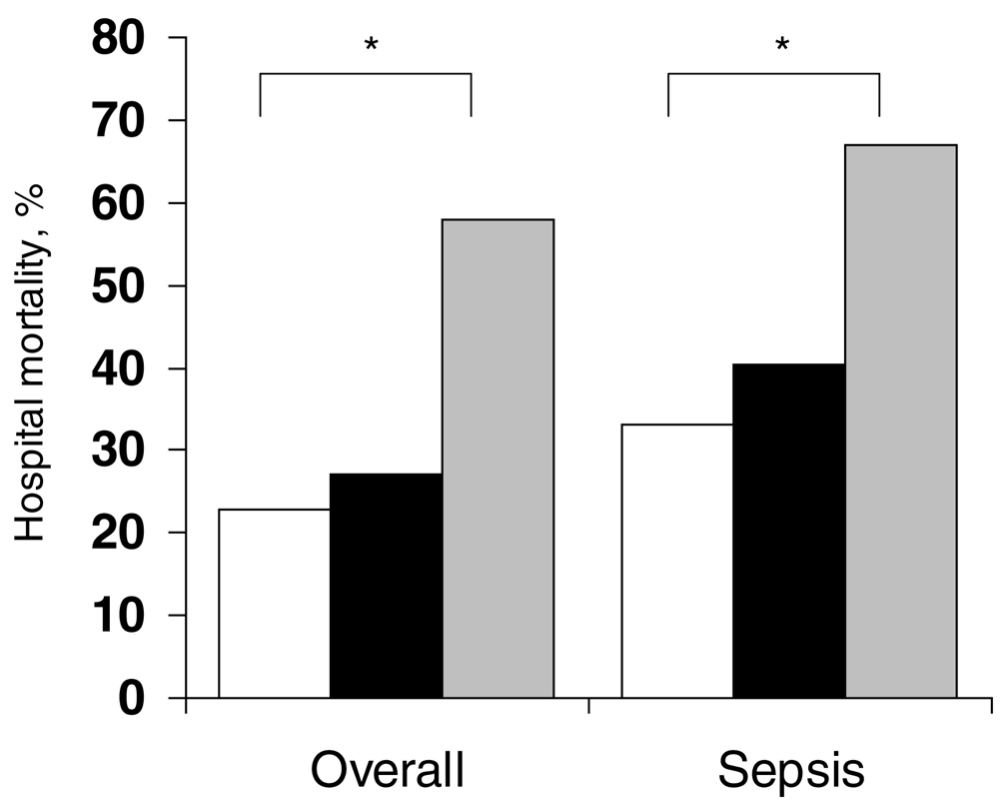
Hospital mortality in the three groups of patients overall and in patients with sepsis. White bars = no cancer; gray bars = haematological cancer; black bars = solid tumours. *p < 0.001 versus no-cancer group.

ICU (20% versus 18%) and hospital (27% versus 23%) mortality rates were similar in patients with solid tumours and those without cancer, respectively, but medical patients had a higher hospital mortality rate than surgical patients (41% versus 21%; p < 0.001). However in multivariable analysis, surgical status was not an independent predictor of mortality in patients with solid cancers. Patients with haematological cancer had higher ICU (42% versus 18%) and hospital (58% versus 23%) mortality rates than non-cancer patients (both p < 0.001) (Figure 2). The same pattern was present when only the patients with sepsis were analysed in the three groups (Figure 3).

In a multivariable analysis, in the patients with solid tumours, SAPS II score, sepsis, ALI/ARDS and mechanical ventilation were associated with increased hospital mortality (Table 4). In patients with haematological cancer, SAPS II score and ALI/ARDS were associated with increased hospital mortality (Table 5).

**Table 4:** Prognostic factors for hospital mortality by multivariate forward stepwise logistic regression analysis in patients with solid cancer (n = 404)

	**OR**	**95% CI**	**p value**
SAPS II*	1.07	1.05 to 1.08	<0.001
**Sepsis**	2.1	1.2 to 3.7	0.01
**ALI/ARDS**	2.5	1.2 to 5.3	0.014
**Mechanical ventilation**	2.4	1.2 to 4.7	0.015

ALI = acute lung injury; ARDS = acute respiratory distress syndrome; CI = confidence interval; OR = odds ratio; SAPS = simplified acute physiology score. *on admission.

Hosmer and Lemeshow goodness-of-fit test chi-squared = 10.15 (p = 0.26). This model has a 79.5% correct classification (50.9% for non-survivors and 90.3% for survivors).

**Table 5:** Prognostic factors for hospital mortality by multivariate forward stepwise logistic regression analysis in patients with haematological cancer (n = 69)

	**OR**	**95% CI**	**p value**
SAPS II*	1.07	1.0. to 1.2	0.002
**ALI/ARDS**	5.3	1.4 to 20.4	0.015

ALI = acute lung injury; ARDS = acute respiratory distress syndrome; CI = confidence interval; OR = odds ratio; SAPS = simplified acute physiology score. *on admission.

Hosmer and Lemshow goodness-of-fit test chi-squared = 15.53 (p = 0.1). This model has a 75.4% correct classification (80.0% for non-survivors and 69.0% for survivors).

## Discussion

This study showed that 15% of patients admitted to European ICUs have cancer (mostly solid tumours). Previous studies described only oncological patients in specialised ICUs [4-6] or were based on retrospective analyses of patients admitted to a single centre without comparison with a non-cancer population [1,10,22]. Analysis of a large US database of more than seven million adult hospital admissions showed that only 9% of admissions were associated with a diagnosis of cancer [23]; however, no specific data were presented on ICU admissions. Overall in our study, the outcome of patients with solid cancer was comparable with that of the general ICU population, with a 27% hospital mortality rate. However, in patients with more than three organs failing, more than 75% of those with cancer died compared with 50% of patients without cancer.

We report our results separately for patients with solid and haematological malignancies as these populations are quite different [10]. Patients with haematological cancers were more severely ill and more commonly had sepsis than patients without cancer, resulting in the highest ICU and hospital mortality rates. The poor prognosis of patients with haematological malignancies who require ICU admission has been well documented, with global hospital mortality rates of 45 to 55% [22,24], increasing to 72% when mechanical ventilation is required [25]. However, recent reports have stressed that aggressive treatment of critical illness events, as well as starting chemotherapy in the ICU for a life-threatening malignancy-related complication, can be lifesaving even when infection or organ failure is present [26].

In contrast, patients with solid tumours had similar severity scores and general profiles to the non-cancer population; they were somewhat older and more commonly had sepsis, factors associated with a worse outcome, but they were more commonly surgical admissions, a factor generally associated with a better outcome than medical admissions [27].

The ICU mortality rate for cancer patients in our study is lower than that previously reported [28]; however, a direct comparison is difficult because of the lack of data on the origin of cancer in our study and the possibility that less 'aggressive' malignancies could have been included. More recent papers have reported ICU mortality rates of 40 to 69% [22,24,29,30]; a lower mortality rate of just 10% was reported in one study but half of the patients were admitted for uncomplicated monitoring [31].

The intensity of treatment was the same in cancer patients as in the general population, as shown by the similar use of mechanical ventilation, vasoactive agents and haemofiltration. Patients with solid tumours were less likely to be monitored with a pulmonary artery catheter, and this was not explained by the differences in heart surgery or by the higher frequency of cardiac failure.

Sepsis is one of the major causes of ICU admission for cancer patients and is an important cause of hospital mortality and morbidity. Cancer has been reported in about 17% of medical admissions associated with sepsis [18,32], with a higher incidence in patients with haematological cancer, probably because of associated leucopenia [33]. Indeed, infection was the main cause of admission for these patients (52%) in our study with a predominance of respiratory infections, as reported previously [17,34,35]. Apart from a higher incidence of *E. coli *and abdominal infections in patients with solid tumours than in non-cancer patients (which could not be explained by the larger number of surgical admissions in solid tumour patients or by the incidence of surgical wound infections), we found a similar spectrum of micro-organisms in patients with and those without cancer. This was even true for infections due to *Candida *species, which are usually more common in leucopenic cancer patients [36]. ICU-acquired infection rates were also comparable. These observations suggest that these patients can be treated with the same antibiotic protocols as other ICU patients if there is no febrile neutropenia.

A multivariable analysis identified a higher severity score and the presence of ALI/ARDS as independent prognostic factors for hospital mortality in patients with haematological cancers, and a higher SAPS II score, mechanical ventilation, presence of sepsis and presence of ALI/ARDS in solid cancer patients. Acute physiology and chronic health evaluation (APACHE) II [37] and SAPS II [38] scores have been specifically validated in certain groups of critically ill cancer patients. The SOFA score also has good prognostic value in critical haemato-oncological disease, suggesting that outcome for ICU cancer patients is determined primarily by the organ dysfunctions induced by complications rather than by the stage of the underlying malignancy [12,39,40]. Our study confirms that survival is dependent on the number of organ failures and that respiratory insufficiency, especially when mechanical ventilation is required [13,41-43], is associated with the highest risk of death.

A limitation of our study, which was not focused specifically on cancer patients, is that we had no specific information about the characteristics of the cancer, including type, stage, histological findings, anticancer treatments or performance status. The defined groups of 'solid' and 'haematological' cancers encompass different diseases with different biological behaviours and severities, thus we could not correlate mortality to these characteristics. However, in the ICU setting, the physiological changes induced by the acute illness may represent the major determinant for the outcome of patients, more than cancer-related characteristics [4]. In addition, the group of cancer patients with more than three organs failing was small and conclusions on the influence of organ dysfunction on mortality should be made with caution. Finally, decisions to limit therapy, and particularly 'do not resuscitate' orders, were not recorded.

## Conclusions

The interesting aspect of our study was the inclusion of consecutive admissions of cancer and non-cancer patients during the same, albeit limited, time period. This study can be seen as an audit of clinical practice in Europe concerning the admission of patients with cancer to the ICU, the intensity of treatment and the types of complications. Thus, our results have ethical implications. Malignancies are becoming increasingly common, especially as the population ages, and cancer patients are likely to represent an increasing proportion of ICU populations. As the mortality rate in patients with cancer in our study was similar to that reported in recent studies and cancer patients underwent complete resuscitation and monitoring, our observations suggest that patients with a poor functional status or refractory malignancy are not being admitted to the ICU; treatment of critical complications resulted in acceptable rates of ICU mortality, without evidence of futile therapy. Similar to previous observations [3,13,38], our study emphasises that ICU admission should not be denied only on the basis of a patient having a neoplastic disease.

## Key messages

• Fifteen percent of patients admitted to European ICUs have cancer.

• ICU and hospital mortality rates were similar in patients with solid tumours and those without cancer.

• Our study emphasises that ICU admission should not be denied only on the basis of a patient having a neoplastic disease.

## Abbreviations

ALI: acute lung injury; APACHE: acute physiology and chronic health evaluation; ARDS: acute respiratory distress syndrome; CI: confidence interval; FiO_2_: inspired fraction of oxygen; ICU: intensive care unit; OR: odds ratio; PaO_2_: arterial partial pressure of oxygen; SAPS: simplified acute physiology score; SD: standard deviation; SOAP: Sepsis in Acutely ill Patients; SOFA: sequential organ failure assessment.

## Competing interests

The authors declare that they have no competing interests.

## Authors' contributions

JLV conceived the initial SOAP study. AA, CS, RM, YS and JLV participated in the design and coordination of the SOAP study. YS performed the statistical analyses. FT and JLV drafted the present manuscript. All authors read and approved the final manuscript.

## Supplementary Material

Additional file 1 A Word file listing participants in the Sepsis Occurrence in Acutely Ill Patients (SOAP) study in alphabetical order. Click here for file
